# Mitochondria-associated membranes in heart failure: from molecular mechanisms to therapeutic targets

**DOI:** 10.3389/fphys.2026.1797296

**Published:** 2026-05-12

**Authors:** Shuning Li, Yanjie Lian, Xiaolei Lai, Juju Shang, Wenlong Xing, Hongxu Liu

**Affiliations:** 1 Beijing Hospital of Traditional Chinese Medicine, Capital Medical University, Beijing, China; 2 Department of Infectious Diseases, Guang’anmen Hospital, China Academy of Chinese Medical Sciences, Beijing, China; 3 Department of Cardiovascular Diseases, Beijing Hospital of Traditional Chinese Medicine, Capital Medical University, Beijing, China

**Keywords:** calcium homeostasis, heart failure, mitochondria-associated endoplasmic reticulum membranes, organelle interaction, therapeutic target

## Abstract

Mitochondria-associated endoplasmic reticulum membranes (MAMs) serve as pivotal functional contact sites linking mitochondria and the endoplasmic reticulum, playing a role in orchestrating various cellular life activities, including calcium homeostasis, mitochondrial quality control, endoplasmic reticulum stress, lipid synthesis and transport, inflammation and innate immunity, apoptosis, autophagy, ferroptosis, and oxidative stress. Recent research has demonstrated that the structural and functional dysregulation of MAMs significantly contributes to the onset and progression of heart failure. This review systematically examined the molecular composition, structural features, and dynamic regulatory mechanisms of MAMs, emphasizing their central roles in the pathophysiological processes of heart failure, such as calcium homeostasis imbalance, mitochondrial dynamics disorders, endoplasmic reticulum stress, metabolic reprogramming, and inflammatory and immune responses. We proposed an in-depth analysis of the differential manifestations of MAMs across distinct heart failure phenotypes (HFrEF and HFpEF) and summarized potential therapeutic strategies targeting MAMs, along with the challenges encountered in their clinical translation. Finally, we proposed a novel research paradigm for MAMs based on multi-omics integration and artificial intelligence, offering a theoretical foundation for the development of precise treatment plans for heart failure.

## 1 Introduction

Heart failure (HF) represents the terminal stage of various cardiovascular diseases, with an estimated global prevalence of 55.4 million patients, imposing a substantial burden on healthcare systems worldwide (Baudry et al., 2025). Despite continuous advances in pharmacotherapy and device-based interventions such as cardiac resynchronization therapy (CRT), HF remains prone to recurrence and its long-term prognosis (Hulot et al., 2025) continues to be unfavorable. Despite advances in guideline−directed medical therapy, the 5−year all−cause mortality after a diagnosis of HF remains as high as 75%. Furthermore, the age−adjusted incidence of HF has stabilized over recent decades, and mortality has paradoxically increased among young adults. These observations suggest that the incremental benefits of current treatment strategies may have reached a plateau, underscoring an urgent need for the identification of novel therapeutic targets (Khan et al., 2024; 2025). Research indicates that microstructural remodeling at the subcellular level, resulting in cell apoptosis, hypertrophy, myocardial fibrosis, and other pathological changes, serves as a pivotal driver in the progression of HF (Gong et al., 2025). Over the past few years, there has been a surge of interest in organelle interactions, with numerous studies highlighting their crucial role in maintaining cellular homeostasis and regulating diverse physiological and pathological processes (Chen et al., 2024). Mitochondria is now recognized not merely as “energy powerhouses” but as multifunctional metabolic-integrative processing systems (MIPS) that integrate ATP synthesis, calcium ion (Ca²^+^) signaling, reactive oxygen species (ROS) generation, and various metabolic reactions, while also serving as signaling hubs that coordinate cellular stress responses and organelle crosstalk via their double-membrane structure (Picard and Shirihai, 2022). The endoplasmic reticulum (ER), on the other hand, serves as the central hub for protein synthesis and modification, lipid synthesis, and calcium homeostasis regulation. A close physical association exists between the outer mitochondrial membrane (OMM) and the ER membrane, known as mitochondrial-associated endoplasmic reticulum membranes (MAMs) (Mohan and Talwar, 2025). As the central hub for inter-organelle communication, MAMs orchestrate crucial cellular processes including calcium signaling, lipid metabolism, and mitochondrial quality control, playing a pivotal role in the onset and progression of diseases such as HF, hypertension, diabetes, and atherosclerosis (Yu et al., 2025). While several reviews have discussed MAMs in cardiovascular disease (Zhang et al., 2023, 2025; Luan et al., 2022; Li et al., 2022), the present review distinguishes itself by (i) proposing a conceptual “Dynamic Change Model” of MAMs remodeling across HFrEF stages, (ii) systematically comparing MAMs dysfunction between HFrEF and HFpEF with recent human data, and (iii) integrating the roles of MAMs in innate immunity and immunometabolic reprogramming as a novel dimension in HF pathogenesis. Despite these advances, the precise mechanisms and therapeutic potential of MAMs in the development of HF remain to be systematically elucidated, particularly with respect to stage−specific and phenotype−specific therapeutic strategies.

## 2 Location, structure and functions of MAMs

### 2.1 Location and structure of MAMs

As early as the 1950s, biologists observed a close connection between mitochondria and ER when examining rat liver cells under an electron microscope (Bernhard and Rouiller, 1956). It wasn’t until 1990 that Vance successfully isolated MAMs using gradient centrifugation and subcellular fractionation techniques, thereby laying the groundwork for modern MAMs research (Vance, 1990). Subsequently, with the advent of techniques such as super-resolution microscopy and proximity labeling omics, the intricate structure and dynamic regulatory mechanisms of MAMs have been progressively unveiled. The complex comprising inositol 1, 4, 5-trisphosphate receptor (IP3R), voltage-dependent anion channel 1 (VDAC1), and 75-kDa glucose-regulated protein (GRP75) serves as the primary conduit for Ca²^+^ transport within MAMs (Guo et al., 2025; de Ridder et al., 2023). Mitochondrial fusion protein 2 (MFN2) is dual-localized to both the outer OMM and the ER, modulating the distance between the ER and mitochondria through GTPase-dependent homotypic interactions, while also orchestrating mitochondrial fusion (Naón et al., 2023; Peñalva et al., 2024). Vesicle-associated membrane protein B (VAPB) resides in the ER, whereas phosphatidylinositol transfer protein 51 (PTPIP51) is situated in the mitochondria; the VAPB-PTPIP51 complex regulates the distance between these two organelles and concurrently governs lipid transport, autophagosome formation, and calcium homeostasis (Mórotz et al., 2022; Yeo et al., 2021). The Fis1-BAP31 complex facilitates caspase-8-mediated apoptotic signaling, while PDZD8 maintains calcium homeostasis, collectively underscoring the diverse functions of MAMs proteins (Liu et al., 2024). Besides structural components, MAMs also incorporate a substantial number of functional regulatory factors that work in concert to orchestrate their biological activities. These regulatory components can be categorized as follows: mediators of Ca²^+^ signaling, such as transient receptor potential vanilloid subtype 1 (TRPV1, which facilitates Ca²^+^ flow from ER to mitochondria) (Tessier et al., 2023); mitochondrial quality control, including dynamin-related protein 1 (DRP1, which regulates mitochondrial fission) and autophagy and beclin 1 regulator 1 (AMBRA1, which governs mitochondrial autophagy) (Duan et al., 2023; Strappazzon et al., 2020); and cell apoptosis, exemplified by Bcl-2-associated X protein (Bax, which modulates OMM permeabilization) (Li et al., 2024; Popgeorgiev and Gillet, 2022). Collectively, these components empower MAMs to regulate cellular metabolism and participate in processes like cell death. The complexity of MAMs’ functions also arises from their unique lipid composition, with cholesterol (CHOL) levels 40-60% higher than those in most ER regions, and an abundance of phosphatidylethanolamine (PE), phosphatidylserine (PS), and sphingolipids. These lipids collectively form lipid raft microdomains, facilitating protein aggregation and signal transduction cascades, thereby establishing MAMs as a pivotal hub for lipid-mediated cellular regulation (Bernal et al., 2023; Fernandes et al., 2023, 2024). A defining feature of MAMs is their dynamic nature, evident at multiple levels (Wu et al., 2023): structural (ER-mitochondrial distances tunable between 10–80 nm), compositional (rapid proteome remodeling under stress), and functional (instantaneous regulation of calcium flux and lipid transport) (Carter et al., 2022; Sonn et al., 2021; Yepuri et al., 2023). For instance, autophagy stimulation can trigger the translocation of cardiolipin (CL) from the mitochondrial inner membrane to MAMs, where it interacts with AMBRA1 to facilitate autophagosome formation (Manganelli et al., 2021, 2021). Metabolites like glucose, glutamine, and free cholesterol can swiftly adjust the contact area and spacing of MAMs (Sun et al., 2024). This dynamic nature allows MAMs to precisely orchestrate cellular metabolism and respond to stress, yet it also suggests that MAMs dysfunction may disrupt cellular homeostasis through various pathways (Mao et al., 2022; Prinz et al., 2020). In chronic conditions such as HF, persistent neurohormonal activation and oxidative stress can induce structural damage and compositional alterations in MAMs, resulting in calcium homeostasis imbalance, energy metabolism disorders, and cell death. Hence, comprehending the dynamic regulatory mechanisms of MAMs is vital for devising targeted therapeutic strategies.

### 2.2 Functions of MAMs

**Figure 1:**
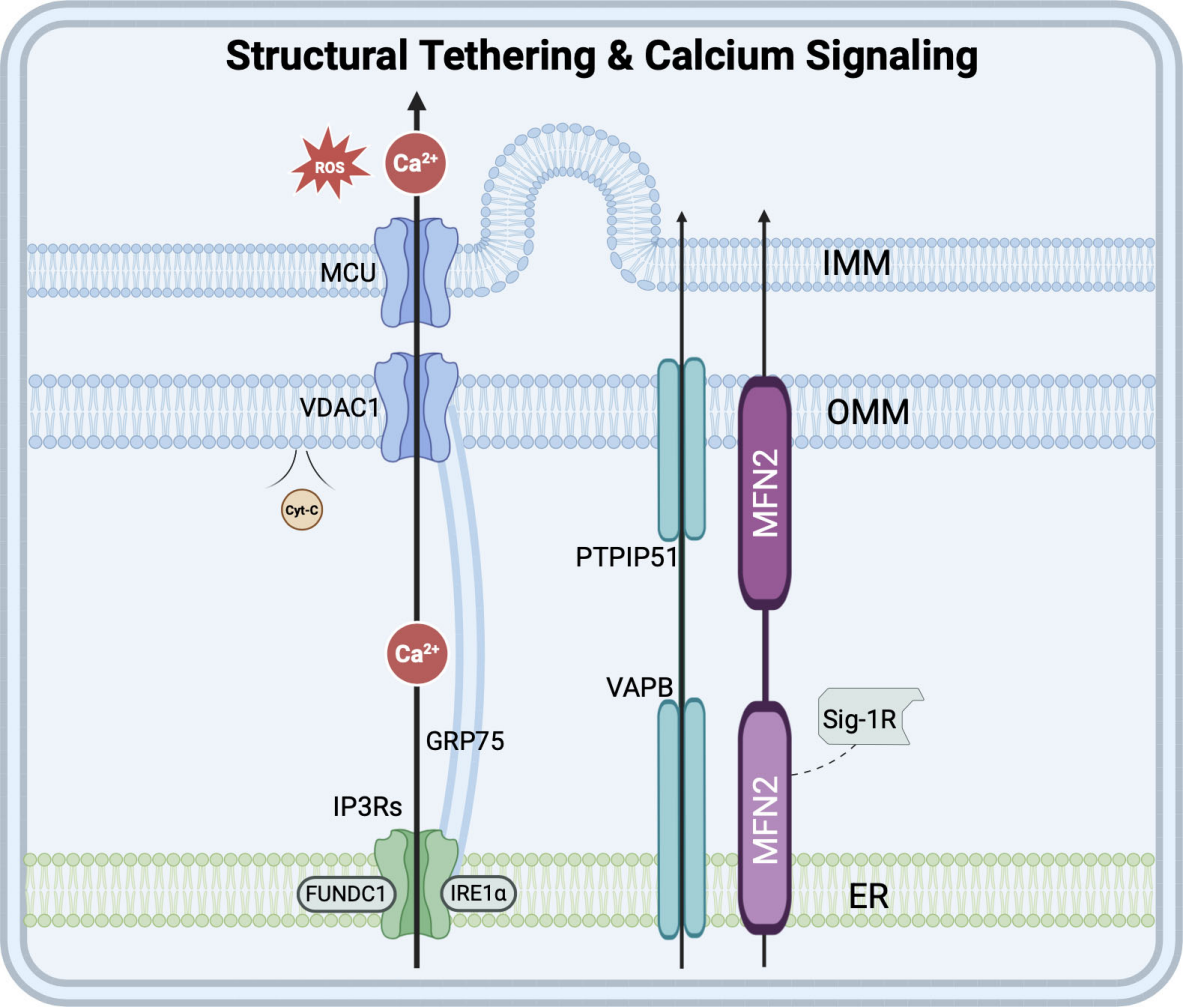
Structural tethering and calcium signaling at MAMs. The IP3Rs-GRP75-VDAC1-MCU axis mediates Ca²^+^ transfer from ER to mitochondria. Tethering is maintained by VAPB-PTPIP51 and MFN2 complexes. FUNDC1 and IRE1α interact with IP_3_R to stabilize calcium signaling architecture. Excessive mitochondrial Ca²^+^ triggers ROS production and mPTP opening. HDL-C uptake via SCARB1 and ORP5/8-mediated PS transfer are also depicted. (Created using Biorender).

**Figure 2:**
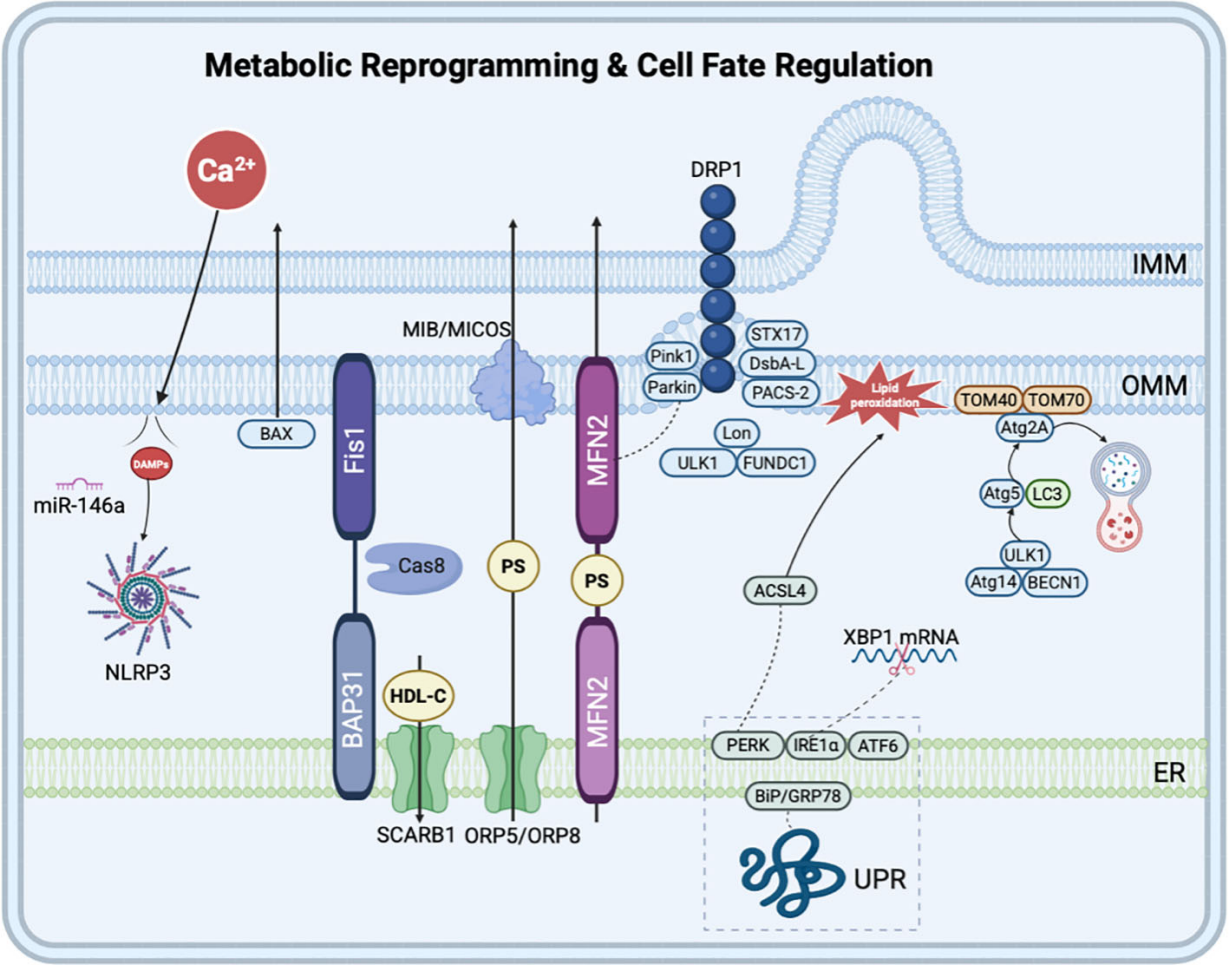
MAMs in metabolic reprogramming and cell fate regulation. MAMs serve as platforms for autophagosome formation (ULK1, Atg14, BECN1), mitophagy (PINK1-Parkin, FUNDC1-ULK1), and mitochondrial fission (DRP1 recruitment by STX17, DsbA-L, PACS-2). Apoptosis is triggered via Fis1-Bap31-Caspase-8 and Bax translocation. ER stress activates the UPR through PERK, IRE1α, and ATF6α. Ferroptosis is promoted by PERK-ACSL4-mediated lipid peroxidation. NLRP3 inflammasome assembly and inflammatory miRNA enrichment also occur at MAMs. (Created using Biorender).

MAMs establish dynamic protein-lipid interfaces through mutual contact and interaction between the ER and mitochondria, facilitating direct material exchange and signal communication between organelles (Peng et al., 2025; Resende et al., 2022). MAMs play a pivotal role in regulating cellular physiological processes and maintaining metabolic homeostasis, including calcium homeostasis, mitochondrial quality control, and ER stress, among others (Hong et al., 2025) (**Figures 1**, **2**).

#### 2.2.1 Calcium homeostasis

Ca²^+^ serves as a crucial signaling molecule within cells, with the ER and mitochondria playing pivotal roles in maintaining intracellular calcium homeostasis (Maione et al., 2022). MAMs act as a platform for Ca²^+^ transport between the ER and mitochondria, orchestrating their interactions to prevent cytoplasmic or mitochondrial Ca²^+^ overload or deficiency (Tangeda et al., 2022). Within MAMs, the IP3R-GRP75-VDAC1 complex forms the primary conduit for Ca²^+^ transfer from ER to the mitochondria, a process detailed further in the context of HF pathophysiology below (Nandwani et al., 2024; Zhang et al., 2024; Zhu et al., 2023). Several proteins within MAMs are associated with the IP3R-GRP75-VDAC1 complex. For instance, inositol-requiring enzyme 1α (IRE1α) and FUN14 domain-containing protein 1 (FUNDC1) function as scaffold proteins in MAMs, physically interacting with IP3R to regulate Ca²^+^ flux between the ER and mitochondria. The efficiency of Ca²^+^ transport channels in MAMs is maximized when the distance between the ER and mitochondria is from 20nm to 25nm (Lotlikar et al., 2025). Studies indicate that the length and tightness of connections of MAMs tethering proteins, such as VAPB-PTPIP51, may be critical factors influencing Ca²^+^ transport (Obara et al., 2024). Notably, this interaction is particularly prominent in cardiomyocytes, where the contact area between interfibrillar mitochondria (IFM) and the sarcoplasmic reticulum (SR, the specialized ER in muscle cells) is significantly larger than in most other cell types. In contrast, subsarcolemmal mitochondria (SSM) exhibit less extensive ER contact. Perinuclear mitochondria (PNM), which cluster around the nucleus, exhibit distinct MAM characteristics compared to IFM and SSM, with specialized roles in regulating nuclear calcium signaling and gene expression responses to metabolic stress (Fernandez-Sanz et al., 2014). During each contraction cycle, the Ca^2+^ released by the ER is swiftly absorbed by mitochondria via MAMs, instantly activating mitochondrial metabolic enzymes. This enables ATP production to synchronize with mechanical contraction demands on a millisecond scale, achieving a precise balance between energy supply and consumption. Consequently, MAMs play a pivotal role in excitation-contraction coupling (ECC).

#### 2.2.2 Mitochondrial quality control

Mitochondrial quality control encompasses processes such as mitochondrial fission, fusion, and autophagy, which collectively maintain a relative equilibrium in mitochondrial quantity, structure, and function (Wang et al., 2022). During the initial stage of mitochondrial fission, synaptic fusion protein 17 (STX17), disulfide bond A oxidoreductase-like protein (DsbA-L), and phosphofuran acid cluster sorting protein 2 (PACS-2) within MAMs recruit DRP1 to these sites (Li, Li et al., 2022). The DRP1 protein then assembles into a helical structure and cleaves the mitochondria, thereby completing mitochondrial fission (Tábara et al., 2025). As the initiation site for mitochondrial division, MAMs underscore their pivotal role in cellular homeostasis and are particularly vital for cells with high energy demands, such as cardiomyocytes and neurons. MFN2 interacts with other MAMs components, including Sig-1R, to facilitate MFN2 oligomerization and promote mitochondrial fusion. MAMs also play a critical role in promoting mitochondrial autophagy by activating both ubiquitin-dependent and non-ubiquitin-dependent pathways (Ponneri et al., 2025; Sun et al., 2022).

#### 2.2.3 ER stress

When misfolded and unfolded proteins accumulate in the ER lumen, cells experience ER stress (ERS), triggering the unfolded protein response (UPR) to restore cellular homeostasis (Yu et al., 2020). Moderate ERS can restore normal ER function through UPR, whereas prolonged ERS may result in cell death. Under normal physiological conditions, PERK, IRE1α, and activated transcription factor 6 (ATF6) within the ER lumen bind to BiP/GRP78. However, the accumulation of a substantial quantity of unfolded proteins within the ER and their subsequent binding to BiP/GRP78 can induce the dissociation and release of PERK, IRE1α, and ATF6α, thereby initiating the UPR (Halder et al., 2022). IRE1α aggregates within MAMs and upregulates the expression of folding and degrading enzymes through the splicing of XBP1 mRNA, facilitating the degradation of misfolded proteins (Jia et al., 2024). The ERS receptor PERK interacts with other proteins in the MAMs region, inhibiting overall protein synthesis and reducing the influx of nascent proteins into the ER (Jeong et al., 2021).

#### 2.2.4 Lipid synthesis and transport

Lipids play a pivotal role in regulating a myriad of life processes. The ER and mitochondria serve as the primary hubs for eukaryotic membrane biogenesis and rely on the lipid synthesis and transport functions of MAMs (Bassot et al., 2023; Szabo et al., 2023). MAMs create a hydrophilic microenvironment between the ER and mitochondria, facilitating the bidirectional, non-vesicular transport of phospholipids (Liu et al., 2023). MFN2 specifically binds to PS and transports it to the mitochondria. Additionally, the oxysterol-binding protein-related proteins ORP5 and ORP8, localized in MAMs, can also mediate PS transport by interacting with the intermembrane space bridging complex (MIB/MICOS) and the OMM protein PTPIP51 (Monteiro-Cardoso et al., 2022). The formation and activation of MAMs are directly influenced by CHOL levels, which, in turn, regulate the CHOL metabolic balance of the entire cell (Montesinos et al., 2024). When extracellular high-density lipoprotein cholesterol (HDL-C) is internalized and transported to the ER via SCARB1, it triggers the formation and activation of MAMs. When CHOL is in excess on the cell membrane and transported back to the ER, it accumulates in the MAMs region. MAMs downregulate the activity of SREBP2, a key regulatory factor for cholesterol synthesis, and degrade cholesterol synthases (such as HMGCR) through the resident protein ERLIN2 (Manganelli et al., 2021).

#### 2.2.5 Inflammation and innate immunity

Inflammation serves as a vital mechanism for the body to defend against damage and infection, while chronic inflammatory responses can damage cells and tissues, leading to a variety of diseases. MAMs are increasingly recognized as a central platform connecting energy metabolism and immune sensing, and their dysfunction profoundly influences the metabolic reprogramming of immune cells and the intensity of inflammatory responses (Rieusset, 2018). MAMs serve as an indispensable molecular platform for the assembly and activation of the NOD-like receptor family pyrin domain-containing protein 3 (NLRP3) inflammasome, a role that is particularly critical in immune cells. Under resting conditions, NLRP3 is primarily localized to both mitochondria and the ER. When cells are subjected to stress (e.g., metabolic disorders, pathogen infection), MAMs integrate multiple upstream signals and become the central hub for NLRP3 inflammasome assembly. Ca²^+^ transferring from the ER to mitochondria regulated by MAMs, along with the production of mitochondrial reactive oxygen species (mtROS), are key initiating signals for NLRP3 activation. Ca²^+^ signaling promotes conformational changes in NLRP3 by acting on NLRP3 itself or its upstream regulatory molecules (Pereira et al., 2022; Li et al., 2023). Upon activation, NLRP3 and its adaptor protein, apoptosis-associated speck-like protein containing a CARD (ASC), translocate to and enrich at MAMs (Ni et al., 2023). Studies have confirmed that under pathological conditions such as sepsis, deubiquitination and translocation of NLRP3 to MAMs are critical steps in inflammasome activation (Li et al., 2026). MAMs not only serve as simple scaffolds but can also form higher-order complexes. For example, promyelocytic leukemia protein (PML) localizes to MAMs and forms a trimeric complex with NLRP3 and the purinergic receptor P2X7R, thereby precisely regulating inflammatory responses within the tumor immune microenvironment. Pathogen infection can modulate immune responses by regulating MAMs-associated proteins. For instance, Mycobacterium tuberculosis infection upregulates the expression of the mitochondrial fusion protein MFN2 to promote efficient assembly of the NLRP3 inflammasome (Xu et al., 2020). Furthermore, the SARS-CoV-2 N protein drives MAMs-mediated mitochondria-ER stress crosstalk via the lncRNA NEAT1, inducing metabolic reprogramming and hyperinflammation in macrophages (Qing et al., 2026). Additionally, MAMs are enriched with various inflammation-responsive microRNAs (e.g., miR-146a, miR-142-3p, and miR-142-5p) (Wang et al., 2020), which can locally regulate the translation of MAMs proteins, thereby finely tuning the intensity of inflammatory responses. In the context of cardiovascular disease, mitochondrial dynamics and MAMs function in macrophages are closely interrelated. By regulating the metabolic switch of macrophages from oxidative phosphorylation to glycolysis (i.e., immunometabolic reprogramming), MAMs influence their polarization toward pro-inflammatory (M1) or anti-inflammatory (M2) phenotypes, thereby determining the outcome of cardiovascular diseases such as atherosclerosis (Zhong et al., 2025). In summary, MAMs act as a critical signaling hub connecting cellular metabolic status and innate immune responses. Their dysfunction directly drives chronic inflammatory cycles, which are important drivers of myocardial fibrosis and ventricular remodeling in diseases such as HF.

#### 2.2.6 Others

Furthermore, MAMs are closely associated with processes such as apoptosis, autophagy, ferroptosis, and oxidative stress. The transfer of Ca²^+^ from the ER to mitochondria is crucial for maintaining mitochondrial function; however, excessive Ca²^+^ accumulation can induce mitochondrial calcium overload and trigger apoptosis (Larrañaga-SanMiguel et al., 2025; Rosa et al., 2022). MAMs serve as a pivotal platform (Herrera-Cruz et al., 2021) for autophagosome formation, aggregating various autophagy-related proteins, such as autophagy-related gene 14 (Atg14), Atg5, and BECN1, to jointly regulate the formation and maturation of autophagosomes (Koyama-Honda and Mizushima, 2022; Yuan et al., 2020). Additionally, MAMs participate in the ferroptosis process by modulating Ca²^+^ transport (Guo et al., 2024; Liu et al., 2025; Zhang et al., 2024) and lipid metabolism (Fang et al., 2021; Sassano et al., 2025; von Krusenstiern et al., 2023). ROS is an indispensable substance for maintaining cellular homeostasis. Under mild stress conditions, mitochondrial generation of ROS triggers membrane lipid peroxidation. Glutathione (GSH) and GPX4 within the ER can reduce lipid free radicals to harmless lipid alcohols, thereby mitigating oxidative stress within the mitochondria (Shiiba et al., 2025). However, under persistent stimulation, the ER transports excessive Ca²^+^ to the mitochondria, leading to damage.

## 3 MAMs and HF

### 3.1 Ca^2+^ disorder and dysfunction of contraction/relaxation

Under physiological conditions, MAMs act as the central platform for ER-mitochondrial calcium transport, efficiently shuttling Ca²^+^ stored in the ER to the OMM via the IP3R-GRP75-VDAC1 complex (Huo and Molkentin, 2024). Subsequently, Ca²^+^ enters the mitochondrial matrix through MCU, activating pyruvate dehydrogenase (PDH) and α-ketoglutarate dehydrogenase (KGDH). This process generates NADH and FADH2, ultimately producing ATP through oxidative phosphorylation (Boyman et al., 2020; Gunter et al., 1994). Nevertheless, the concentration of mitochondrial Ca²^+^ must be precisely regulated: a moderate increase (ranging from 50 to 200 nM) can stimulate respiration and enhance ATP production. However, excessive accumulation (>500 nM) induces mitochondrial calcium overload, triggering a burst of ROS production, mPTP opening, mitochondrial swelling, and ultimately cell death (Lai and Qiu, 2020). MAMs help maintain mitochondrial Ca²^+^ within safe limits by regulating the contact area between the ER and mitochondria, as well as the expression levels of the IP3R-GRP75-VDAC1 complex.

Although MAMs dysfunction is implicated in both HFrEF and HFpEF, the underlying biological characteristics of MAMs differ substantially between these two phenotypes. In HFrEF, particularly of ischemic origin, MAMs exhibit dynamic structural remodeling during disease progression. During the acute injury stage of HFrEF as observed in post-infarction rat models, such as 1–7 days, the contact area of MAMs increases, the distance between the ER and mitochondria shortens, and the expression of IP3R-GRP75-VDAC1 is upregulated, leading to mitochondrial calcium overload and an explosive increase in ROS production (Wan et al., 2023; Wang et al., 2022). Excessive ROS triggers lipid peroxidation and mPTP opening, releasing pro-apoptotic factors that activate the caspase-9/3 cascade and drive cardiomyocyte apoptosis (Hohorst et al., 2025; Li et al., 2024). Oxidative damage to both mitochondrial DNA and nuclear DNA accelerates the aging of myocardial cells and activates the poly (ADP-ribose) polymerase 1 (PARP-1) pathway, depleting nicotinamide adenine dinucleotide (NAD+) reserves. This leads to energy metabolism disorders and impairs the efficiency of myocardial excitation-contraction coupling (Harrington et al., 2023). Concomitant ROS-mediated inhibition of SERCA2 and NCX1 further impairs Ca²^+^ extrusion, exacerbating cellular calcium overload and contractile dysfunction (Hinton et al., 2024). During the compensatory stage of HF (1–4 weeks), the myocardium initiates adaptive remodeling. Quantitative ultrastructural analyses in preclinical models indicate that this phase is characterized by a 10–20% reduction in the relative MAMs contact area (defined as the fraction of the outer mitochondrial membrane in close apposition with the ER) and a widening of the ER–mitochondrial gap from the physiological range of 10–25 nm to approximately 30–40 nm (Luan et al., 2021; Li et al., 2022). Notably, this structural loosening is not merely a preclinical phenomenon: recent transmission electron microscopy analyses of human myocardial tissue from patients with dilated, hypertrophic, and ischemic cardiomyopathy have demonstrated a consistent increase in the distance between the sarcoplasmic reticulum and mitochondria, accompanied by smaller and more rounded mitochondrial morphology across all HF etiologies (Latchman et al., 2025). This observation has been interpreted as a potential protective negative feedback mechanism that limits excessive calcium flux into mitochondria during the initial insult. However, it remains to be determined whether this loosening of ER--mitochondrial contacts represent a directed adaptive response or is merely a passive consequence of cytoskeletal remodeling and organelle swelling during the transition from acute injury. The upregulation of the MICU1 regulatory protein raises the threshold for mitochondrial Ca²^+^ uptake, thereby mitigating mitochondrial and cellular damage. However, in the chronic stage of HF (>4 weeks), prolonged neurohormonal activation and oxidative stress result in structural damage to MAMs, downregulation of key proteins such as MFN2 and PINK1, weakened ER-mitochondrial coupling, insufficient mitochondrial Ca²^+^ uptake, decreased TCA cycle activity, and reduced ATP production (Garbincius and Elrod, 2021; Liu et al., 2021; Xiao et al., 2025). This “Dynamic Change Model of MAMs” elucidates the transition of energy metabolism from a compensatory to a decompensated state during the clinical progression of HF, and offers a theoretical foundation for stage-specific treatment strategies: excessive coupling of MAMs should be suppressed during the acute injury stage, for instance, by employing MCU inhibitors or IP3R antagonists; the structural integrity of MAMs should be preserved during the compensatory stage, with avoidance of excessive intervention; and in the chronic stage, the stability of MAMs should be enhanced to restore energy metabolism. For instance, Sigma-1 receptor agonists, such as PRE-084 or the clinically available antidepressant fluvoxamine, have been shown to stabilize MAMs structure and confer cardioprotective effects. Furthermore, the small-molecule compound CP2 has been demonstrated to restore mitochondrial fusion and ER–mitochondria tethering by activating MFN2, thereby improving energy metabolism in the failing heart (Garbincius and Elrod, 2022).

While the above model primarily reflects the pathological trajectory observed in HF with reduced ejection fraction (HFrEF) triggered by ischemic injury, HF is a heterogeneous syndrome with distinct underlying mechanisms across different phenotypes. Unlike ischemic HFrEF, the functional impairment of MAMs in patients with HF with HFpEF is predominantly characterized by metabolic remodeling rather than structural disruption (Garbincius et al., 2020). Transcriptomic analysis of human myocardial biopsies from diabetic patients with aortic stenosis and preserved ejection fraction revealed downregulation of mitochondrial calcium signaling pathways, including MAMs-related genes such as MICU1, accompanied by reduced VDAC-IP3R proximity and altered metabolic gene expression (Cherpaz et al., 2024). Complementary single-cell transcriptomic analyses of human hypertrophic hearts have further demonstrated that MAMs-related proteins preferentially accumulate in cardiomyocytes at the initial stage of hypertrophy, followed by a gradual decline that synchronizes with cardiomyocyte subtype switching. Notably, weighted gene co-expression network analysis revealed that MAMs-related genes cluster into a module specifically correlated with diabetic cardiomyopathy, providing a transcriptomic basis for the distinct MAMs dysfunction observed in metabolism-driven HFpEF compared with ischemia-driven HFrEF (Luan et al., 2023). This also suggests that therapeutic strategies targeting MAMs in HFpEF should prioritize enhancing metabolic flexibility rather than solely modulating calcium signaling.

**Figure 3:**
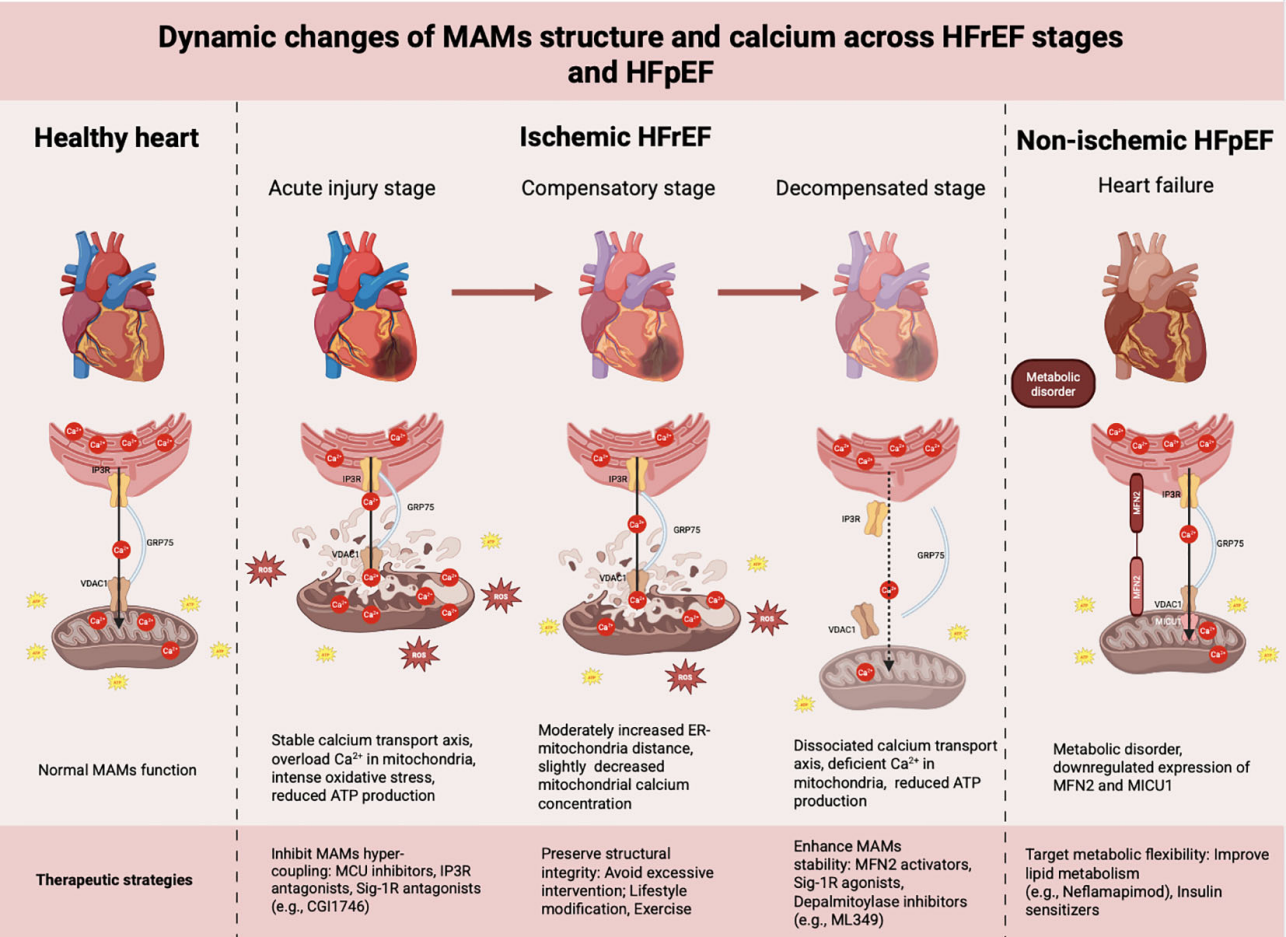
Dynamic changes of MAMs’ structure and calcium/energy metabolism across HFrEF stages and HFpEF. Ischemic HFrEF: Under physiological conditions, the ER transports Ca²^+^ to mitochondria via the IP3R-GRP75-VDAC1 pathway. During acute injury, the distance between the ER and mitochondria shortens, resulting in a massive influx of Ca²^+^ into mitochondria, which triggers oxidative stress, mitochondrial swelling and rupture, and a consequent reduction in ATP production. In the compensatory stage, the ER-mitochondrial distance increases slightly, accompanied by a mild decrease in mitochondrial Ca²^+^ levels and alleviated oxidative stress. During decompensation, the distance between the ER and mitochondria increases significantly, the IP3R-GRP75-VDAC1 complex dissociates, mitochondrial Ca²^+^ concentration drops, and ATP levels decline sharply. Non-ischemic HFpEF: Under the influence of metabolic disorders and other stimuli, the expression of MFN2 and MICU1 decreases. Proposed stage-specific therapeutic interventions targeting MAMs dynamics are annotated at the bottom of each corresponding phase to illustrate potential translational strategies. (Created using Biorender).

Based on these predominantly preclinical observations, we propose a conceptual “Dynamic Change Model of MAMs” to describe the trajectory of ER-mitochondrial communication during the progression of HF. This framework hypothesizes that MAMs undergo a biphasic shift—from acute pathological hyper-coupling to chronic adaptive hypo-coupling—which may parallel the clinical transition from a compensated to a decompensated state in terms of energy metabolism. It is important to note that this model is derived largely from rodent studies and *in vitro* cardiomyocyte assays. Direct longitudinal validation in human myocardial tissue is currently lacking. Furthermore, this dynamic remodeling appears to be distinct in HFpEF, where metabolic comorbidities rather than ischemic cascades dominate MAMs dysfunction. Therefore, while this model offers a theoretical foundation for staged therapeutic interventions, it should be regarded as a working hypothesis that requires rigorous future validation (**Figure 3**). It is important to distinguish this model from the well−established concept of the “metabolic switch” in HF. The classical metabolic switch describes a shift in substrate preference—from fatty acid oxidation toward increased reliance on glycolysis—as the failing heart attempts to maintain ATP production under stress. While this paradigm explains the biochemical reprogramming of energy pathways, it does not address the spatial and structural determinants that govern the efficiency of substrate delivery, calcium−dependent dehydrogenase activation, or organelle communication. In contrast, the Dynamic Change Model of MAMs proposed here emphasizes the evolving physical and functional architecture of ER–mitochondria contact sites. Specifically, it delineates how MAMs transition from acute hyper−coupling (which drives calcium overload and oxidative damage) to chronic hypo−coupling (which impairs mitochondrial calcium uptake and oxidative capacity). This model thus offers a subcellular−level mechanistic framework that complements, rather than contradicts, the metabolic switch hypothesis. It explains not only why the switch occurs but also when and how the structural integrity of MAMs dictates the reversibility of metabolic dysfunction. Consequently, the MAMs−centric model provides a basis for stage−specific interventions that target organelle interface dynamics, a dimension that is not captured by traditional views of metabolic remodeling alone.

### 3.2 Mitochondrial dynamics and myocardial remodeling

Several mitochondrial dynamic proteins associated with MAMs have been confirmed to influence the progression of heart disease. In a mouse model of FARS2 deficiency, its deficiency activates DRP1, leading to excessive mitochondrial fission, fragmentation, loss of function, and overproduction of mtROS, consequently resulting in reduced ATP generation. mtROS not only directly damages cells but also serves as signaling molecules to activate signaling pathways promoting hypertrophy (e.g., MAPK/ERK) and fibrosis (e.g., TGF-β) (Lozhkin et al., 2022), ultimately causing myocardial hypertrophy and fibrosis (Li et al., 2024). Under pressure overload in rodent models, under sustained pressure overload, downregulation of MFN2 disrupts the physical tethering of MAMs. This leads to a pathologically significant increase in intermembrane distance (>50 nm) (Giacomello and Pellegrini, 2016), which effectively uncouples the functional ER–mitochondria axis beyond the threshold required for efficient Ca^2+^ transfer. Consequently, mitochondrial dynamics shift toward excessive fission, resulting in fragmentation and bioenergetic failure that drive maladaptive myocardial remodeling (Li et al., 2025; Li et al., 2022; Ren et al., 2022). These studies indicate that disturbances in mitochondrial dynamics can lead to myocardial remodeling, and targeting mitochondrial dynamics may emerge as a novel strategy for preventing and treating HF-related myocardial remodeling (Luan et al., 2022).

### 3.3 ERS and myocardial remodeling

Recent research has underscored the significance of ERS (such as the PERK pathway) in the development of myocardial hypertrophy and the progression of HF. NOGO-B, a member of the ER reticular protein family, plays a crucial role in preserving the reticular morphology of the ER and exerts a negative regulatory influence on ER-mitochondrial contacts. Inhibiting NOGO-B facilitates myocardial hypertrophy and the activation of cardiac fibroblasts through the activation of the PERK/ATF4 signaling pathway and the ATF6-mediated ERS pathway (Gao et al., 2020; Li et al., 2018). This highlights the potential of targeting ERS to ameliorate myocardial remodeling in HF treatment.

### 3.4 Disorder of lipid anabolic metabolism and metabolic reprogramming

Metabolic reprogramming in HF represents one of its fundamental pathological characteristics, evidenced by the transition of myocardial cells from highly efficient fatty acid β oxidation (yielding 106 ATP per palmitic acid molecule) to less efficient glycolysis (producing merely 2 ATP per glucose molecule), coupled with a decline in mitochondrial oxidative phosphorylation efficiency and an overproduction of ROS (Jia et al., 2025). Under stress conditions, a continuous influx of Ca²^+^ into mitochondria via MAMs results in mitochondrial calcium overload. This, in turn, damages key dehydrogenases (PDH, IDH, α-KGDH) and F_1_F_0_-ATP synthase within the tricarboxylic acid cycle, triggers the opening of the mPTP, collapses membrane potential, dissipates the proton gradient, halts oxidative phosphorylation entirely, and causes a sharp decline in ATP synthesis. Consequently, cells resort to the less efficient glycolytic pathway to sustain the most basic ATP supply. Beyond this quantitative energetic shortfall, MAMs dysfunction impairs metabolic flux at a qualitative level by uncoupling substrate availability from enzymatic activity. The physical proximity maintained by MAMs creates Ca²^+^ microdomains that lower the activation threshold for key mitochondrial dehydrogenases, particularly PDH and α-KGDH. In the context of chronic HF, structural loosening of MAMs—characterized by increased ER-mitochondrial distance and downregulation of tethering factors such as MFN2—effectively desensitizes the mitochondrial matrix to ER-derived Ca²^+^ signals. Consequently, despite adequate cytosolic substrate supply, the TCA cycle operates below its maximal oxidative capacity due to suboptimal Ca²^+^-dependent activation of PDH and KGDH. This mechanism provides a direct subcellular explanation for the clinical observation that the failing heart exhibits impaired oxidative metabolism even when circulating free fatty acids and glucose remain abundant. Furthermore, impaired lipid synthesis and transport functions in MAMs reduce the efficiency of substrate delivery, such as fatty acids, to mitochondria, leading to cytoplasmic accumulation of fatty acids. This reduction in substrate delivery efficiency is further compounded by MAMs-dependent dysregulation of mitochondrial membrane lipid composition. MAMs serve as the primary conduit for non-vesicular transport of PS from the ER to mitochondria, a process mediated by the ORP5/8 and PTPIP51 complexes. Within mitochondria, PS is decarboxylated to PE, a critical structural lipid required for the optimal assembly and stability of respiratory chain supercomplexes (I+III+IV). Disruption of MAMs integrity therefore not only impedes fatty acid import but also compromises the very infrastructure of the electron transport chain, thereby reducing the maximal oxidative capacity specifically for fatty acid-derived substrates. This lipid-mediated component of MAMs dysfunction explains why interventions that merely increase fatty acid uptake often fail to improve cardiac energetics in HF, as the downstream oxidation machinery remains structurally compromised. These fatty acids are then esterified into triglycerides or converted into toxic lipids like ceramides and diacylglycerols, creating a lipotoxic environment that inhibits mitochondrial oxidative capacity for both fatty acids and glucose, thereby promoting a shift towards glycolytic metabolism (Gallo et al., 2024; Beaulant et al., 2022). Studies in mouse models have demonstrated that the multifunctional protein DsbA-L, localized in the mitochondrial matrix and MAMs, can stabilize or modulate lipid transport/synthesis-related proteins (e.g., ATGL, FATP1) on MAMs through its oxidoreductase activity or chaperone function. This facilitates the orderly β-oxidation of fatty acids from lipid droplets to mitochondria, preventing the intracellular accumulation of free fatty acids and subsequent metabolic reprogramming (Oniki et al., 2020; Chen et al., 2019). In addition to lipid metabolism disorders, MAMs dysfunction can further exacerbate the energy crisis in cardiomyocytes by affecting amino acid metabolism. Studies have shown that dysfunctional MAMs suppress the activity of the branched-chain ketoacid dehydrogenase (BCKD) complex, leading to the abnormal accumulation of branched-chain amino acids (BCAAs) and their metabolic intermediates (such as branched-chain α-keto acids) within the mitochondria of cardiomyocytes. These accumulated metabolites subsequently inhibit the activity of pyruvate dehydrogenase (PDH)—a key enzyme linking glycolysis and the tricarboxylic acid (TCA) cycle. The inhibition of PDH directly blocks the aerobic oxidation of glucose, thereby further reinforcing the shift from oxidative phosphorylation to glycolysis as the primary energy substrate metabolism in cardiomyocytes (Du et al., 2022). Concurrently, MAMs dysfunction is often accompanied by aberrant activation of the mTOR signaling pathway and impairment of the insulin-PI3K-Akt pathway, which reduces the translocation of glucose transporter GLUT4 to the cell membrane and further compromises glucose uptake and utilization in cardiomyocytes. In summary, under conditions of MAMs dysfunction, cardiomyocytes face not only impaired fatty acid oxidation and lipotoxicity (as described above) but also a “multiple-hit” scenario characterized by blocked aerobic glucose oxidation and reduced glucose uptake. Together, these metabolic defects compel cardiomyocytes to rely primarily on inefficient glycolysis for ATP production, ultimately leading to a severe state of “energy starvation” that directly drives the progression of HF (Acevedo et al., 2024).

Among the two types of HF, the pathophysiological mechanisms underlying HFpEF are closely linked to systemic metabolic disorders, often accompanied by comorbidities such as obesity and diabetes. These conditions lead to damage in the insulin signaling pathway, persistent activation of pro-inflammatory cytokines, and excessive production of ROS, which can disrupt MAMs function (McCandless et al., 2022), causing an imbalance in intracellular calcium homeostasis, particularly delayed calcium ion reuptake during diastole. Furthermore, the accumulation of toxic lipid metabolites damages cell membrane structure and signal transduction, resulting in reduced efficiency of mitochondrial oxidative phosphorylation and an ROS burst (Heidenreich et al., 2022). The energy metabolism substrate preference in cardiomyocytes shifts from primarily oxidizing long-chain fatty acids to relying more on anaerobic glycolysis, leading to a significant reduction in ATP production and placing the heart in an “energy-starved” state during diastole. Consequently, ATP deficiency and the associated molecular events (Kumar et al., 2019), such as conformational changes in titin and increased myofilament calcium sensitivity, collectively form the metabolic basis for impaired active relaxation and increased passive stiffness in the myocardium, directly contributing to the functional phenotype of HFpEF (Fayyaz et al., 2025).

The clinical relevance of MAMs dysfunction in lipid metabolism is further substantiated by studies in dilated cardiomyopathy (DCM), a leading cause of HF. Disruption of MAMs in DCM initiates pathological cascades including ER stress, inflammation, and cell death, ultimately destabilizing cellular homeostasis and contributing to contractile dysfunction. Identifying MAMs as key modulators of cardiac health may therefore provide novel insights for early diagnosis and targeted therapies in DCM and other HF subtypes (He et al., 2025).

### 3.5 Inflammation, innate immunity and myocardial fibrosis

During the onset and progression of HF, the inflammatory response in myocarditis is inextricably linked to fibrosis (Rao et al., 2021). Damage-associated molecular patterns (DAMPs), generated by initial insults such as ischemia, pressure overload, or metabolic disorders, serve as key triggers of sterile inflammation. MAMs function as a critical sensing platform for DAMP signals and a spatial hub for NLRP3 inflammasome assembly (Xue et al., 2022). Specifically, MAMs’ structural dysregulation leads to aberrant endoplasmic reticulum–mitochondrial calcium transfer, resulting in mitochondrial calcium overload and dysfunction, which in turn precipitates an mtROS burst and mtDNA leakage—the primary upstream source of DAMPs within the failing myocardial microenvironment. These mitochondria-derived DAMPs are efficiently presented within MAMs-enriched microdomains, where they specifically activate the NLRP3 inflammasome in cardiomyocytes and macrophages. As a key platform for NLRP3 assembly and activation, MAMs integrate signals such as Ca²^+^ flux, ROS production, and mitochondrial dysfunction to promote inflammasome activation. The activated NLRP3 inflammasome mediates caspase-1-dependent cleavage, leading to the maturation and release of potent pro-inflammatory cytokines IL-1β and IL-18, thereby initiating chronic low-grade inflammation within the myocardium (Missiroli et al., 2023). This inflammatory signaling directly triggers the pathological transition toward fibrotic remodeling. Activated IL-1β significantly upregulates the expression of profibrotic factors such as TGF-β, TNF-α, and IL-6 through activation of downstream pathways including NF-κB. These factors drive the phenotypic conversion of cardiac fibroblasts into myofibroblasts, resulting in excessive deposition, disordered arrangement, and enhanced cross-linking of extracellular matrix (ECM) components, primarily type I collagen, thereby inducing myocardial fibrosis (Yamada et al., 2025) and subsequently impairing both active myocardial relaxation and contractile function (Li et al., 2022).

**Figure 4:**
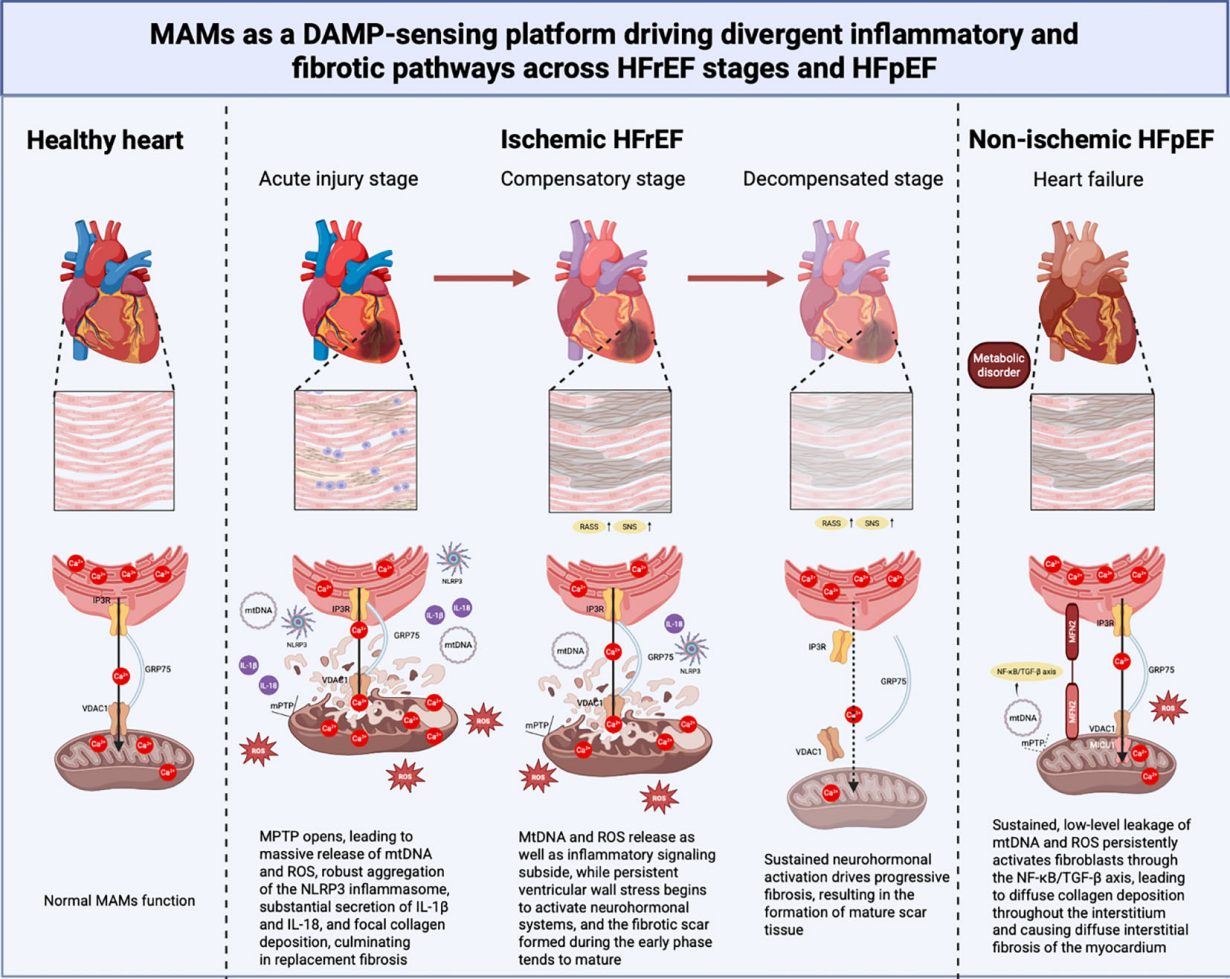
MAMs as a DAMP-sensing platform driving divergent inflammatory and fibrotic pathways across HFrEF stages and HFpEF. Ischemic HFrEF: During acute injury, MAMs hyper-coupling induces severe mitochondrial calcium overload and mPTP opening, leading to massive release of mtDNA and ROS, robust aggregation of the NLRP3 inflammasome, substantial secretion of IL-1β and IL-18, and focal collagen deposition, culminating in replacement fibrosis. In the compensatory stage, mtDNA and ROS release as well as inflammatory signaling subside, while persistent ventricular wall stress begins to activate neurohormonal systems, and the fibrotic scar formed during the early phase tends to mature. During decompensation, sustained neurohormonal activation drives progressive fibrosis, resulting in the formation of mature scar tissue. Non-ischemic HFpEF: Sustained, low-level leakage of mtDNA and ROS persistently activates fibroblasts through the NF-κB/TGF-β axis, leading to diffuse collagen deposition throughout the interstitium and causing diffuse interstitial fibrosis of the myocardium. (Created using Biorender).

Notably, this process exhibits distinct regulatory patterns across HF subtypes and across the different stages of HFrEF progression. In the acute stage of HFrEF, tight MAMs coupling induces massive mitochondrial calcium overload and mPTP opening, leading to a burst release of mtDAMPs and a robust caspase-1/IL-1β inflammatory storm. This not only directly exacerbates cardiomyocyte death but also rapidly initiates focal replacement fibrosis. In the compensatory stage, as MAMs undergo adaptive loosening and the mitochondrial calcium uptake threshold is elevated, mtDAMP release and inflammatory signaling partially subside. However, the fibrotic scar formed during the acute phase matures during this period, while persistent ventricular wall stress begins to activate neurohormonal systems, setting the stage for subsequent decompensation. In the decompensated stage, MAMs structures become further dissociated, with disassembly of the IP3R-GRP75-VDAC1 complex, and insufficient mitochondrial calcium uptake leads to energetic collapse. Although the acute burst of mtDAMPs has subsided, sustained activation of the RAAS and sympathetic nervous system maintains a low-grade pro-inflammatory and pro-fibrotic microenvironment, driving progressive ventricular remodeling and worsening systolic dysfunction. As for the HFpEF, persistent metabolic disturbances (e.g., obesity, diabetes) lead to looser MAMs’ structures and low-grade oxidative stress. Sustained, low-level leakage of mtDAMPs persistently activates fibroblasts through the NF-κB/TGF-β axis, resulting in diffuse interstitial fibrosis and increased ventricular wall stiffness, which further impairs coronary microcirculatory perfusion, culminating in a clinical phenotype dominated by diastolic dysfunction. Concurrently, diffuse fibrosis and microvascular dysfunction disrupt normal nutrient and signal exchange between cardiomyocytes and the interstitium. These alterations continuously activate neuroendocrine systems, including the RAAS and the sympathetic nervous system, which in turn further exacerbate inflammation and fibrosis, thereby establishing an intractable vicious cycle (Thorp and Filipp, 2025). Therefore, MAMs serve not only as upstream regulators of sterile inflammation but also as a central pathological hub that transduces DAMP signals into myocardial fibrosis and HF progression. The dynamic evolution of MAMs in HFrEF—characterized by an evolving trajectory from acute inflammatory storm through compensatory adaptation to chronic pro-fibrotic remodeling, stands in striking contrast to the persistent low-grade inflammation driving diffuse fibrosis in HFpEF, underscoring the potential of MAMs as a subtype-specific therapeutic target in HF.(**Figure 4**).

## 4 Targeting MAMs for HF treatment

Given the pivotal role of MAMs in the progression of HF, targeting MAMs has emerged as a potential therapeutic strategy for HF. Precise regulation of MAMs offers a fresh perspective for developing interventions against HF (**Table 1**). It is noteworthy that the mechanisms underlying the treatment of HF by targeting MAMs through Ca²^+^ signaling regulation must be examined in light of the varying Ca²^+^ demands of mitochondria at different stages of HF. For ischemic HFrEF, during the acute injury stage, it is imperative to inhibit excessive coupling between the ER and mitochondria, disrupt the connecting proteins within MAMs, and thereby reduce mitochondrial Ca²^+^ concentration. Conversely, in the decompensated stage, enhancing MAMs’ stability, promoting Ca²^+^ influx into mitochondria, restoring mitochondrial respiration, and increasing ATP energy supply are essential to alleviate energy deprivation in myocardial cells. Evidence from animal studies suggests that lifestyle modifications, encompassing dietary adjustments and moderate aerobic exercise, represent viable approaches to improving MAMs structure and function. However, human data remain scarce. Dysfunction of MAMs induced by a high-fat diet serves as a critical factor in promoting cell apoptosis (Chen et al., 2022). Conversely, a low-fat, healthy diet can ameliorate MAMs structure and function by regulating lipid metabolism and calcium homeostasis (Wang et al., 2021). For instance, ω-3 fatty acids can influence calcium homeostasis, mitochondrial function, inflammatory responses, and antioxidant activity by modulating MAMs structure and function. Exercise training can safeguard the structural integrity and function of MAMs through various mechanisms, including upregulating MFN2, reinforcing and stabilizing MAMs structure, laying the groundwork for normal Ca²^+^ and lipid exchange, stabilizing the IP3R-GRP75-VDAC1 complex, regulating Ca²^+^ flux, enhancing glucose and substrate oxidation, boosting ATP production, activating the FUNDC1 and PINK1/Parkin pathways, and facilitating the efficient recognition and clearance of damaged mitochondria at MAMs. Moreover, exercise itself serves as a potent stimulus for mitochondrial biogenesis (Allen et al., 2025; Liu et al., 2025). However, the precise efficacy and safety of these interventions in treating HF necessitate further validation through clinical trials. Additionally, the intricate structure and dynamic nature of MAMs pose significant challenges for drug development: the presence of thousands of associated proteins limits the feasibility of achieving strict target specificity (Lu et al., 2022). MAMs exhibit varying, and sometimes even opposing, roles across different stages of HF, necessitating tailored treatment strategies. Many resident proteins of MAMs lack exclusive subcellular localization, complicating spatial targeting and often leading to off-target effects from interventions. The profound complexity arises from the intricate interplay between the structural components of MAMs and their enzymatic effects, particularly evident in critical processes like phospholipid exchange. The potential compensatory and competitive interactions among proteins such as ORP5/8, MFN2, and CDS2 present formidable obstacles to drug design (Xu et al., 2022). Calcium channel modulators exhibit a certain degree of toxicity. Besides, conventional calcium channel blockers (e.g., L-type calcium channel antagonists) or mitochondrial calcium uniporter (MCU) inhibitors often lack spatial selectivity for MAMs-localized channels such as IP_3_R and VDAC1. Systemic administration may inadvertently suppress physiological calcium signaling in non-cardiac tissues or even compromise cardiac contractility, precipitating hypotension, bradycardia, or atrioventricular block. Moreover, complete blockade of mitochondrial calcium uptake can paradoxically impair ATP production and exacerbate energy starvation in the failing heart, underscoring the narrow therapeutic window of these agents. Consequently, the development of MAMs-restricted calcium modulators—or alternative strategies that normalize rather than abolish ER-mitochondrial Ca²^+^ transfer—remains a critical yet unmet need for clinical translation (Zhao and Sheng, 2025).

**Table 1:** Drugs or compounds targeting MAMs and their mechanisms of action.

Therapeutic target	Applicable HF period	Name of drugs and compounds	Action mechanism	Developmental stage
Ca^2+^ signal regulation	Acute injury stage	Polypeptide TAT-FUNDC1-S (Wang et al., 2021)	Competitively binding to IP3R to block the normal interaction between endogenous FUNDC1 protein and IP3R, thereby disrupting the structure of MAMs.	Small animal models
		Palmitoyltransferase inhibitors, such as 2-BP, CMA, etc.	Inhibiting the ZDHHC enzyme family to reduce the palmitoylation and localization of specific proteins on MAMs, weakening excessive coupling of MAMs, and consequently alleviating mitochondrial calcium overload.	*In vitro*/cellular
		CGI1746	Antagonizing the molecular chaperone protein Sig-1R of MAMs to block the transfer of Ca^2+^ from the ER to mitochondria.	Small animal models
	Decompensation stage	Depalmitoylase inhibitors, such as ML349, C83, C115, etc (He et al., 2023).	Inhibiting depalmitoylating enzymes (such as APT1 and APT2) to enhance the palmitoylation of MAMs-related proteins, thereby tightening the connections of MAMs.	*In vitro*/cellular
Lipid synthesis and transport		Neflamapimod (Larrea et al., 2025)	Improving the phospholipid synthesis function of damaged MAMs, protecting mitochondrial membrane structure, reversing PDH inhibition, and restoring energy metabolism.	Clinical trials (phase II for neurological indications; cardiovascular studies preclinical)
Inflammation and innate immunity		MAO inhibitors (Deshwal et al., 2018)	Reducing MAO-dependent ROS production to preserve ER-mitochondria homeostasis, prevent mast cell degranulation, and improve diastolic function in diabetic cardiomyopathy	Small animal models
Mitochondrial quality control		Mdivi-1 (Cherubini et al., 2020)	Inhibiting DRP1 to prevent excessive mitochondrial fission and maintain the structural integrity of MAMs.	*In vitro*/cellular
ERS		Thymoquinone (Li et al., 2025)	Directly binding to and stabilizing the mitochondrial protein ATAD3A to maintain the stability of MAMs structure and alleviate ERS during I/R.	Small animal models
		Nanoparticle Se@LNT (Tan et al., 2025)	Alleviating mitochondrial calcium overload and ERS.	Small animal models
		1-DNJ (1-Deoxynojirimycin) (Xing et al., 2025)	Modulating the PERK-ATF4/MFN2 signaling pathway to alleviate oxidative stress-induced apoptosis and potentially maintaining ER-mitochondria communication	*In vitro*/cellular
		MitoQ (Souza-Neto et al., 2022)	Mitochondria-targeted antioxidant that scavenges mtROS, alleviates MAMs dysfunction, and modulates ER stress by improving mitochondrial function	Clinical trials (phase II for cardiovascular indications)
Ferroptosis		CGI1746	Promoting the accumulation of triglycerides containing polyunsaturated fatty acids to inhibit ferroptosis.	Small animal models

Despite the growing preclinical evidence supporting MAMs as therapeutic targets, clinical translation remains in its infancy. A recent comprehensive review by Zhang et al. highlighted that MAMs have great potential as therapeutic targets for various cardiovascular diseases, including HF, atherosclerosis, and diabetic cardiomyopathy, yet emphasized that the feasibility of incorporating MAMs into diagnostic strategies and treatment plans requires further validation (Zhang et al., 2025). Similarly, Hinton et al. noted that mitochondria–ER contact sites represent “often-neglected factors” in human HF and identified them as promising clinical mitochondrial targets. These analyses underscore both the promise and the current limitations of MAMs-targeted approaches. Future efforts should focus on developing stage-specific interventions that account for the dynamic remodeling of MAMs across the HF trajectory, as well as non-invasive biomarkers (e.g., circulating MAMs-derived miRNAs or imaging-based contact site quantification) to enable patient stratification and treatment monitoring. Future research should integrate multi-omics approaches (including single-cell transcriptomics, spatial proteomics, and metabolomics) with artificial intelligence techniques (such as machine learning for predicting drug-target interactions and deep learning for analyzing dynamic images of MAMs) to elucidate the regulatory networks of MAMs and expedite the clinical translation of MAMs-targeted therapies.

A fundamental obstacle to translating MAMs-targeted therapies is the ubiquitous expression of key tethering proteins such as IP3R, VDAC1, and MFN2 across multiple organ systems. Systemic pharmacological inhibition risks perturbing calcium handling in neurons, insulin secretion in pancreatic β-cells, or mitochondrial dynamics in skeletal muscle. Achieving cardiac-restricted modulation will therefore require innovative targeting strategies. Adeno-associated virus (AAV) vectors, particularly serotypes 9 and 6, exhibit robust cardiotropism and have been successfully employed in clinical trials for gene therapy in HF. Cardiac-specific promoters (e.g., α-MHC, cTnT) further restrict transgene expression to cardiomyocytes. For small molecules, nanoparticle-based delivery systems functionalized with cardiac-homing peptides (e.g., CSTSMLKAC, PCM-1) or antibody fragments recognizing cardiomyocyte surface markers (e.g., anti-TnI) offer promising avenues to concentrate payloads within the myocardium (Weng et al., 2024). Additionally, prodrugs engineered for activation by cardiomyocyte-enriched enzymes (e.g., creatine kinase) may provide an additional layer of spatial control. While these technologies remain largely at the preclinical stage for MAMs applications, their development represents a critical prerequisite for the safe clinical translation of MAMs-directed interventions.

## 5 Research techniques related to MAMs

Due to their nanoscale dimensions, dynamic changes, and functional complexity, MAMs place extremely stringent demands on research methodologies. In recent years, the convergence of a range of interdisciplinary technologies has been driving a fundamental transformation in this field. Cutting-edge approaches such as Fluorescence Resonance Energy Transfer/Fluorescence Lifetime Imaging (FRET/FLIM), Spatiotemporally Resolved Proximity Labeling Technology (Split-TurboID), and Orthogonal Dual-Labeling Technology (OrthoID) have propelled the research paradigm of MAMs from static component analysis to dynamic functional analysis, from spatial proximity inference to direct interaction evidence, and from single-modal descriptions to system-integrated modeling. These technological advancements have not only markedly enhanced detection specificity, spatiotemporal resolution, and result reliability but also empowered researchers to capture protein interactions in live cells in real time, label dynamic proteomic changes within contact microdomains, and leverage artificial intelligence for multidimensional data fusion and mechanistic modeling. Consequently, they have systematically elucidated the dynamic regulatory networks governing the structure and function of MAMs, laying a solid foundation for a deeper understanding of their roles in cellular signal integration and metabolic regulation. (**Table 2**).

**Table 2:** Functions and limitations of relevant research techniques of MAMs.

Type	Name	Function	Limitations
Traditional imaging and morphological analysis	Conventional fluorescence microscope	Labeling organelles with broad-spectrum markers and conducting long-term imaging of live cells are straightforward procedures.	Due to the optical diffraction limit (~200 nm), it is impossible to resolve nanoscale MAMs structures, and only co-localization speculation can be made.
	Electron microscope (with continuous block-face scanning) (Zhou et al., 2020)	It offers sub-nanometer resolution, enabling intuitive visualization of MAMs morphology, precise measurement of membrane spacing, and 3D reconstruction for analyzing dynamic structural changes.	Sample preparation is complex, precluding live cell imaging; it is challenging to correlate functional information of specific proteins, resulting in limited functional insights.
	Fluorescence co-localization analysis (such as Manders coefficient) (Jiang et al., 2024)	It exhibits high sensitivity to the co-localization of specific protein pairs, facilitating quantitative analysis of pixel overlap.	The results are vulnerable to interference from imaging noise, defocused light, and spontaneous fluorescence, potentially leading to false positive signals; direct interactions cannot be proven.
High resolution and dynamic monitoring	Super-resolution microscopes (such as STED) (Damenti et al., 2021; Shen et al., 2020)	By circumventing the diffraction limit (achieving a resolution of several tens of nanometers), it resolves the nanoscale distribution and structural domains of membrane proteins at MAMs.	The imaging speed for live organisms is relatively slow and may fail to capture rapid dynamics; there is a risk of phototoxicity; and the requirements for sample preparation and data analysis are stringent.
	FRET/FLIM (Fluorescence resonance energy transfer/Fluorescence Lifetime Imaging) (Zellmer et al., 2025)	Real-time and quantitative monitoring of close-range protein-protein interactions (<10nm) and microenvironmental changes within MAMs serves as one of the “gold standards” for verifying direct interactions.	There is sensitivity to probe labeling efficiency and spectral crosstalk; instrumentation and data analysis are complex; and fully capturing millisecond-level ultra-fast signal events remains difficult.
New molecular probes and sensors	Split contact point sensor (such as split GFP system) (Elwakiel et al., 2024)	It provides real-time, visual reporting on the formation and dissociation of specific membrane contact sites (e.g., MAMs) in living cells, demonstrating robust dynamic monitoring capabilities.	It usually relies on overexpression, which may disrupt the natural membrane contact state; signal reconstruction is delayed and insensitive to instantaneous rapid changes.
Spatio-temporal resolution omics technology	Proximity labeling technology (such as TurboID, APEX2) (Cho et al., 2020; Lee et al., 2024)	It enables spatiotemporally resolved proteomic analysis in specific regions of living cells to identify the protein composition of MAMs. Split-TurboID: Captures dynamically evolving interaction networks. OrthoID (Orthogonal Dual-Labeling): Simultaneously labels and distinguishes proteins from the OMM and ER membrane, significantly reducing false positives and facilitating the discovery of novel proteins.	There is a possibility of marking transient or extremely closely adjacent non-interacting proteins; endogenous biotin background may cause interference; the labeling reaction may have a slight impact on cellular physiology; and the workload for data validation is substantial.
Data integration and intelligent analysis	AI-driven multimodal integration (Chen et al., 2023)	By integrating multi-scale data from imaging, omics, and dynamic analyses, it achieves high-throughput tracking, automatic feature recognition, and cross-scale correlation analysis of MAMs dynamic processes, overcoming historical analytical limitations.	It relies on high-quality, standardized input data; requires a large amount of annotated data for model training; and the interpretability of algorithms is sometimes inadequate.

Despite the technological advances summarized in **Table 2**, quantifying MAMs structure and dynamics in living cardiomyocytes remains challenging. Transmission electron microscopy (TEM) provides the gold-standard ultrastructural resolution required to measure ER–mitochondrial contact distances (10–80 nm) and has been instrumental in documenting MAMs remodeling in failing human myocardium. However, TEM requires chemical fixation and dehydration, precluding live-cell observation and potentially introducing artifacts in membrane spacing. Moreover, the static nature of TEM images cannot capture the millisecond-to-minute dynamics of contact site formation and dissociation that characterize MAMs function during excitation–contraction coupling. Proximity ligation assays (PLA) offer a complementary approach by detecting protein–protein interactions at MAMs with high specificity in fixed cells and tissues. However, PLA signals represent binary “contact” or “no contact” readouts and lack the spatial resolution to resolve nanoscale distance changes within the physiological 10–25 nm range. Furthermore, PLA cannot provide real-time kinetic information in beating cardiomyocytes, where calcium transients and metabolic demands fluctuate continuously. Emerging techniques such as split-GFP contact site sensors and FRET/FLIM-based proximity reporters have begun to address these limitations by enabling live-cell monitoring of MAMs dynamics. Nonetheless, their application in adult cardiomyocytes—particularly in intact myocardial tissue—remains technically demanding due to challenges in efficient probe delivery, autofluorescence from mitochondrial flavoproteins, and motion artifacts during contraction. Until these methodological barriers are overcome, our understanding of MAMs dynamics in the native cardiac environment will remain largely inferential. A balanced interpretation of MAMs-related findings therefore requires acknowledgment of the inherent constraints imposed by currently available quantification tools.

## 6 Conclusions and prospects

MAMs serve as the core hub for inter-organelle communication, profoundly influencing the pathophysiological processes of myocardial cells, including energy metabolism, contractile function, cell death, and fibrosis, by regulating calcium homeostasis, mitochondrial dynamics, ERS, lipid metabolism, inflammatory responses, and other processes. In different types of HF, such as HFrEF and HFpEF, MAMs exhibit distinct structural and functional dysfunctions, indicating their potential as important biological markers for subtype-specific diagnosis and treatment. Intervention strategies targeting MAMs, including regulating calcium signaling, enhancing MAMs’ stability, and improving lipid metabolism, have demonstrated therapeutic potential in experimental models, offering novel insights for HF treatment. This review extends prior work by providing a stage-specific and phenotype-specific framework for MAMs pathology in HF, integrating emerging evidence on inflammation and metabolism that has not been fully addressed in earlier syntheses. Although the role of MAMs in HF is gradually being elucidated, numerous scientific questions and clinical translation challenges remain. Importantly, as this field matures, it is increasingly necessary to distinguish between well-established mechanistic links and those that remain largely correlative or context-dependent. The IP3R–GRP75–VDAC1 axis governing ER-to-mitochondria calcium flux represents one of the most rigorously validated MAMs-dependent pathways in the cardiovascular system. However, the dynamic change model proposed herein for ischemic HFrEF, while conceptually compelling, is derived predominantly from preclinical models and awaits rigorous validation in human myocardial tissue, particularly across the heterogeneous spectrum of HFpEF. Moreover, a fundamental question persists: does the tightening of ER–mitochondria contacts during acute injury constitute a primary pathological trigger, or is it merely a secondary consequence of organelle swelling and cytoskeletal remodeling? Emerging data from super-resolution microscopy indicate that the relationship between intermembrane distance and calcium transfer efficiency is neither linear nor invariant, cautioning against oversimplified therapeutic strategies aimed solely at loosening or tightening these contacts. Similar interpretive challenges extend to other facets of MAMs biology. The recruitment of DRP1 to MAMs and its role in mitochondrial fission are well documented, yet the pharmacological tools used to interrogate this process, most notably Mdivi-1, exhibit confounding off-target effects on mitochondrial complex I, obscuring the specific contribution of MAMs-localized fission to adverse remodeling. In the context of metabolic reprogramming, the hypothesis that MAMs dysfunction directly instigates the shift from fatty acid oxidation to glycolysis remains speculative; direct *in vivo* evidence that impaired phospholipid transfer at MAMs is sufficient to drive cardiac energetic failure is still lacking. Likewise, while MAMs provide a scaffold for NLRP3 inflammasome assembly, genetic disruption of MAMs tethering proteins invariably alters mitochondrial morphology and mitophagic clearance, rendering it difficult to ascertain whether attenuated inflammation results from loss of the MAMs platform per se or from enhanced removal of damaged, ROS-generating mitochondria. Importantly, while human evidence supporting MAMs dysfunction in HF is accumulating—including ultrastructural analyses of failing human myocardium, single-cell transcriptomic profiling of hypertrophic hearts, and proteomic characterization of HFpEF samples—most findings remain correlative. Prospective longitudinal studies in human cohorts are urgently needed to establish whether MAMs structural and functional changes precede clinical decompensation or merely accompany it. For instance, while the present review primarily focuses on the role of MAMs in cardiomyocytes—the central functional unit of the heart—it is important to acknowledge that other cardiac cell types, including cardiac fibroblasts, endothelial cells, and resident immune cells (e.g., macrophages), also harbor functional MAMs (Kaur et al., 2025). Therefore, future studies should systematically investigate the cell-type-specific roles of MAMs in the failing heart. Current research predominantly focuses on static or single time-point analyses of MAMs, lacking real-time monitoring and mechanistic analysis of their dynamic changes throughout the course of HF. Regarding the “Dynamic Change Model of MAMs” proposed herein for ischemic HFrEF, several caveats must be acknowledged. First, the temporal resolution of MAMs remodeling in human HF remains undefined; the acute, compensatory, and decompensated stages delineated in animal models may not precisely mirror the heterogeneous clinical course in patients. Second, the extent to which altered MAMs proximity acts as a primary driver versus a secondary bystander of metabolic decompensation requires dissection using inducible, cardiomyocyte-specific genetic models. Finally, the generalizability of this model to non-ischemic etiologies or HFpEF is limited, given the divergent metabolic and inflammatory drivers in those conditions. Future studies should integrate live-cell imaging and spatiotemporal omics technologies to uncover the real-time regulatory networks of MAMs during disease progression. While HFrEF and HFpEF exhibit differences in MAMs dysfunction, their molecular underpinnings and metabolic regulatory specificities have not been systematically clarified. Given the complex structure and dense protein interaction networks of MAMs, existing interventions are prone to off-target effects. Therefore, it is essential to develop drug delivery systems with precise subcellular localization and stage-specific adaptability, combined with artificial intelligence to predict drug-target interactions and enhance treatment specificity. Currently, research on MAMs as a target predominantly relies on cell and animal models, with a notable absence of clinical sample validation and prospective cohort studies. The field must now advance beyond descriptive cataloging of MAMs-associated pathways toward rigorous, causality-oriented investigations, which will necessitate the deployment of inducible, cardiomyocyte-specific genetic tools to manipulate MAMs components at distinct disease stages, coupled with advanced live-cell imaging modalities capable of capturing the millisecond-to-minute dynamics of contact site behavior. Distinguishing adaptive from maladaptive MAMs remodeling across the natural history of HF is paramount to avoid the pitfall of therapeutically targeting a compensatory response.

To summarize, as an emerging therapeutic target, MAMs research is now at a pivotal juncture, transitioning from mechanistic exploration to clinical application. Through interdisciplinary collaboration and technological innovation, it is anticipated that personalized HF treatment based on MAMs modulation will be realized in the future, thereby overcoming the current limitations in HF therapy.
